# Acquired Glucocorticoid Resistance Due to Homologous Glucocorticoid Receptor Downregulation: A Modern Look at an Age-Old Problem

**DOI:** 10.3390/cells10102529

**Published:** 2021-09-24

**Authors:** Lee-Maine L. Spies, Nicolette J. D. Verhoog, Ann Louw

**Affiliations:** Department of Biochemistry, Stellenbosch University, Van de Byl Street, Stellenbosch 7200, South Africa; 18244017@sun.ac.za (L.-M.L.S.); nverhoog@sun.ac.za (N.J.D.V.)

**Keywords:** glucocorticoid receptor alpha, glucocorticoids, glucocorticoid receptor dimerization, acquired glucocorticoid resistance, ubiquitin-proteasome system

## Abstract

For over 70 years, the unique anti-inflammatory properties of glucocorticoids (GCs), which mediate their effects via the ligand-activated transcription factor, the glucocorticoid receptor alpha (GRα), have allowed for the use of these steroid hormones in the treatment of various autoimmune and inflammatory-linked diseases. However, aside from the onset of severe side-effects, chronic GC therapy often leads to the ligand-mediated downregulation of the GRα which, in turn, leads to a decrease in GC sensitivity, and effectively, the development of acquired GC resistance. Although the ligand-mediated downregulation of GRα is well documented, the precise factors which influence this process are not well understood and, thus, the development of an acquired GC resistance presents an ever-increasing challenge to the pharmaceutical industry. Recently, however, studies have correlated the dimerization status of the GRα with its ligand-mediated downregulation. Therefore, the current review will be discussing the major role-players in the homologous downregulation of the GRα pool, with a specific focus on previously reported GC-mediated reductions in GRα mRNA and protein levels, the molecular mechanisms through which the GRα functional pool is maintained and the possible impact of receptor conformation on GC-mediated GRα downregulation.

## 1. Introduction

The pharmacological use of glucocorticoids (GCs) can be traced back over a century to the work of the clinical physician, Solomon Solis-Cohen, who reported on the therapeutic benefits of orally administered adrenal gland extracts in the treatment of asthma [1]. However, due to the previously reported bronchodilator effect of adrenaline, these benefits were assumed to be a result of adrenaline from the adrenal medulla [2,3]. Later, however, Thomas Addison would also report on the therapeutic benefits of adrenal extracts; this time, in the treatment of chronic fatigue, muscular degeneration, weight loss and darkening of the skin, in what would later be called Addison’s disease [4,5]. As the therapeutic potential of adrenal extracts became apparent, the search for its active factor became internationally competitive. In 1946, Edward Calvin Kendall isolated four steroidal compounds from adrenal extracts, one of them, cortisol [6]. Later that year, cortisol was synthesized by Sarett [7] and, three years later, rheumatologist Phillip Hench proved cortisol to be extremely beneficial in the treatment of rheumatoid arthritis [2]. Merck and company then began the commercial production of the synthetic GC, cortisone and, in 1950, Kendall and Hench, together with Swiss biochemist, Tadeus Reichard, received the Nobel Prize in Physiology and Medicine for their work regarding adrenal hormones [2]. This catapulted GCs into the limelight and these synthetic steroids quickly became the mainstay therapeutic choice in the treatment of auto-immune and inflammatory-linked conditions [8,9].

The excitement surrounding this wonder drug would, however, quickly die-down as reports of adverse side-effects [10,11] and GC insensitivity [12,13] arose, specifically in cases were high doses of GCs were used for long periods of time. As GC centered research progressed, it was discovered that cortisol (F) and corticosterone, the main endogenous GC in humans and rodents, respectively, are produced and secreted in response to circadian/ultradian and stress cues, and that, in addition to their anti-inflammatory properties, these steroids also regulate life-sustaining processes, such as growth, development, and metabolism [8,14,15,16]. GCs were found to predominantly regulate these processes via the binding and activation of the cytoplasmic ligand-activated transcription factor (TF), the glucocorticoid receptor alpha (GRα) [17,18,19]. Once bound, the GC-GRα complex regulates the transcription of genes through various activation and repression mechanisms. Generally, direct binding of the GC-GRα complex, as a homodimer, at glucocorticoid response elements (GREs) in the promoter of GC-responsive genes, is associated with transcriptional activation, while transcriptional regulation via the monomeric GC-GRα complex is associated with gene transrepression. The latter occurs via both DNA-dependent and independent mechanisms, with DNA-dependent monomeric GC-GRα signaling occurring via the binding of the complex to negative glucocorticoid response elements (nGREs), and DNA-independent monomeric signaling occurring via tethering of the complex to DNA-bound TFs [20,21]. Additional investigation of GC-GR signaling found that dimer-mediated GRα signaling resulted in the transactivation of genes involved in glucose synthesis and fat metabolism, which would explain the many undesirable side effects that often accompany GC therapy. In contrast, monomeric GRα signaling results in the transrepression of pro-inflammatory genes by disrupting the action of TFs, such as nuclear factor kappa B (NFκB) and activator protein 1 (AP-1) [5,8,22]. Since only the immunosuppressive properties of GCs are clinically desired, GC-centered research quickly shifted to the possible functional separation of monomeric and dimeric GRα signaling. While it soon became apparent that this approach was a gross over-simplification of GRα signaling, the strategy would give rise to the development of selective glucocorticoid receptor agonists (SEGRAs) and selective glucocorticoid receptor modulators (SEGRMs). These ligands, which are collectively referred to as SEGRAMS, were essentially designed to preferentially induce monomeric signaling of the GC-GRα complex, over dimeric signaling, in an effort to retain the potent anti-inflammatory properties of GCs while limiting the many undesirable side effects [8,15,23,24]. While some of these ligands have been somewhat successful in addressing the adverse side-effects of GC therapy [25,26,27], the development of GC-mediated resistance still remains a threat and is under-researched.

Currently, approximately 1% of the population of the United Kingdom and the United States undergo long-term GC treatment, of which 30% experience some degree of GC insensitivity [28]. More specifically, up to 10% of asthma patients, ~30% of rheumatoid arthritis and Crohn’s disease patients, and 90% of chronic obstructive pulmonary disease (COPD) and sepsis patients experience GC resistance [28,29,30]. Various regulatory processes, which modulate either GRα function or GRα expression, are responsible for a decrease in GC sensitivity and, ultimately, GC resistance. Based on the underlying cause, GC resistance may be categorized into two major groups: generalized GC resistance and acquired GC resistance [31,32]. Primary generalized GC resistance or hereditary GC resistance mostly affect all tissue types and is characterized by disruptions in GRα functionality, affecting the subcellular localization, ligand binding, and transactivation ability of the receptor, amongst others. Alterations in GRα function largely stem from mutations and/or polymorphisms of the GR gene (NR3C1 in humans, and Nr3c1 in mice), an increase in non-transcriptionally active GR isoforms (e.g., GRβ, GRγ, GR-A) due to alternative splicing events [33,34], and changes in GC sensitivity due to a pro-inflammatory environment [16,35,36]. Unlike generalized GC resistance, acquired GC resistance or secondary GC resistance, mainly affects specific tissues and/or cells in a biological system and is hallmarked by the homologous down-regulation of the GRα [30,37], rather than disruptions in receptor function. This form of GC resistance occurs more frequently and has been linked to a plethora of psychological and pathological conditions/diseases [38,39,40,41,42]. The molecular mechanisms behind generalized GC resistance are better understood, allowing for more precise diagnostic tools [43]. In contrast, however, the molecular mechanism underlying acquired GC resistance, is relatively unknown and thus, diagnosis of this condition requires more exhaustive diagnostic approaches in order to determine GC sensitivity of specific tissue and/or cells [30,43]. The current review will thus be discussing the major role-players in the homologous downregulation of the GRα pool, with a specific focus on previously reported GC-mediated reductions in GRα mRNA and protein levels, the molecular mechanisms through which the GRα functional pool is maintained and the possible impact of receptor conformation on GC mediated GRα downregulation.

## 2. Acquired GC Resistance

Acquired GC resistance is often brought on by the homologous downregulation of GRα mRNA and protein as a result of an increase in circulating GCs, as a consequence of either, chronic GC therapy or as a side-effect of the pathophysiological processes that accompany the inflammatory disease state, or both. This section briefly discusses the interrelatedness of disease- and treatment associated reductions in GRα mRNA and protein levels, before a more in-depth discussion of the known molecular mechanisms which govern GC-mediated GRα downregulation.

Stress-related reductions in GRα mRNA and protein levels are among the most studied disease-associated modulations of the GRα pool, with a reduction in GRα mRNA and/or protein being noted in various human and rodent studies, in response to pre-/post-natal, physical and psychological stress [39,44,45,46,47]. Factors which influence the extent of the stress-related reduction in the GRα pool include, length of exposure to the stressor, the environment in which the stress occurs and how sensitive the individual is to stress [39,44,45,46]. Many, but not all, stress-related modulations of the GRα pool have been linked to an increase in circulating endogenous GCs [39,47,48], subsequently resulting in the homologous downregulation of GRα as a protective mechanism against the detrimental effects of prolonged stress-related activation of GRα signaling. In addition to a decrease in GC sensitivity, a reduction in the GRα pool has been implicated in the increased susceptibility to various psychological and pathological conditions, such as depression and schizophrenia [49,50]. While GRα expression is extremely diverse in patients suffering from psychological conditions, many display increased activation of inflammatory processes and HPA-axis signaling, resulting in hypercortisolaemia and a decrease in the GRα pool in peripheral tissues [51].

Likewise, a significant number of patients who suffer from auto-immune and inflammatory-linked diseases, such as atopic dermatitis (AD) [41], systemic lupus erythematosus (SLE) [52], steroid resistant Type II asthma [53,54], chronic obstructive pulmonary disease (COPD) [55,56], and adult immune thrombocytopenia (ITP) [42], have reduced GRα mRNA expression, and thus, reduced GC sensitivity within peripheral blood mononuclear cells (PBMCs). Additionally, many patients suffering from auto-immune or inflammatory-linked conditions or cancer [57,58,59], receive GC treatment, thus, it is difficult to distinguish between disease-associated and treatment-associated reductions in the GRα pool, within these groups [60]. It should be noted, however, that GC resistance within these disease groups may occur via factors other than the reduction of GR.

Many studies have noted a reduction in GRα mRNA and protein levels in response to exogenous GC-treatment. More specifically, Dexamethasone (Dex), a potent GRα agonist, has been shown to cause up to a 90% reduction in GRα mRNA and/or protein levels [25,61,62,63,64,65,66,67,68,69], with one study demonstrating the complete downregulation of GRα [64]. Similarly, in vivo and ex vivo studies demonstrate a Dex-induced decrease in the GRα mRNA and/or protein pool in various mouse and rat tissues, with many of these studies correlating with the reduction through an increase in GC-insensitivity [25,65,68,70,71,72]. In addition to Dex, other exogenous GCs (e.g., hydrocortisone [73], triamcinolone acetonide [74] and various prednisolone-based steroids [60,75,76,77,78,79] have also been linked to a decrease in the GRα mRNA and/or protein pool.

**Figure 1:**
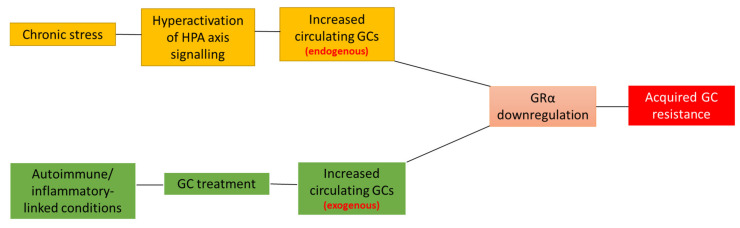
Disease- and treatment-associated effects lead to acquired GC resistance. Disease-associated (orange boxes) triggering of the stress-signaling pathway often results in the dysregulation and subsequent hyperactivation of HPA axis signaling, which, in turn, leads to the overproduction and secretion of endogenous GCs, GRα downregulation and, ultimately, acquired GC resistance. Similarly, the GC-mediated treatment of autoimmune and inflammatory conditions (green boxes), results in an increase in circulating exogenous GCs, which mediates the downregulation of GRα and, subsequently, acquired GC resistance. Complicating matters further, patients suffering from a disease-associated reduction in GRα levels often receive GC-treatment, further compounding the development of acquired GC-resistance. Although the development of acquired GC resistance generally occurs due to a reduction in the GRα functional protein pool, it is important to note that other cellular processes may also be involved in the development of this condition.

In summary, both disease- and treatment-associated reductions in the GRα pool contribute to GC insensitivity and the development of acquired GC resistance (Figure 1). With GC resistance remaining one of the two major drawbacks in GC treatment, it has never been more important to identify and understand the molecular mechanisms involved in the homologous downregulation of GRα.

## 3. Molecular Mechanisms Involved in the Homologous Downregulation of GRα

**Figure 2:**
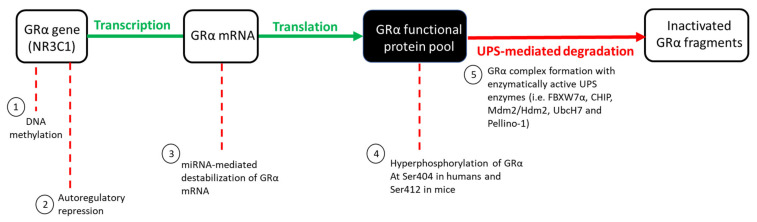
GC-mediated regulation of the GRα functional protein pool. Under basal physiological conditions, GRα synthesis (indicated in green) and degradation (indicated in red) occur at roughly the same rate. An increase in circulating GCs, however, disrupts the equilibrium of these processes, subsequently allowing for an increase in GRα downregulation via various mechanisms: (1) DNA methylation of the GR gene promoter inhibits the initiation of GRα gene transcription, (2) the formation of a repressive autoregulatory loop through which GC-bound GRα decreases the expression of its own nascent mRNA, by forming a long-range interaction with a NCOR1-deacetylase 3-containing repression complex, (3) the binding of miRNAs to AU-rich regions of the mRNA transcript, destabilizes mature GRα mRNA, (4) hyperphosphorylation of the receptor at Ser404 in humans, and Ser412 in mice, serves as a signal for the UPS-mediated degradation of GRα protein which occurs via (5) complex formation between GRα and several UPS enzymes. Hyperphosphorylation of the GRα refers to the phosphorylation of the receptor above that of its basal phosphorylation.

The GRα protein pool, like many other proteins, is regulated at several levels by various molecular mechanisms within living cells. Wilkinson et al. [30,69] eloquently describe the regulation of the GRα pool as a ‘push (synthesis) vs. pull (degradation)’ mechanism. When unperturbed, GRα synthesis and degradation exists in a state of dynamic equilibrium, with these processes occurring at roughly the same rate, thus ensuring that the GRα pool remains constant. GR gene transcription, mRNA translation, and protein synthesis are defined as the ‘push’ of the mechanism, while the ‘pull’ refers to GRα degradation through the ubiquitin proteasome system (UPS) (Figure 2). The dynamic equilibrium state, in which the GRα pool exists under basal physiological conditions, may be perturbed by an increase in circulating GCs (endogenous or exogenous), subsequently increasing the GC-induced downregulation of the GRα functional pool. The GC-mediated regulation of the GRα functional pool includes the epigenetic, transcriptional, post-transcriptional and post-translational regulation of the receptor and occurs in an intricate and specific manner to either stabilize or destabilize the GRα [31,80], with the destabilization of GRα as a potential contributor to acquired GC resistance. While the ligand-independent regulation of GRα levels also occurs [31,32,80], the current review specifically focuses on the molecular mechanism through which GCs mediate the downregulation of the GRα.

### 3.1. GRα mRNA Regulation

Although the GRα is ubiquitously and constitutively expressed, vast heterogeneity exists in the expression of the receptor in different cells and tissues. While this is mostly due to the differential regulation of the GR gene within cells, GCs have been shown to impact the regulation of GRα mRNA at various levels. The following section will thus highlight these GC-mediated effects on GRα epigenetic, transcriptional and post-transcriptional regulation.

#### 3.1.1. Epigenetic Regulation

DNA methylation of the GR gene promoter is one of the mechanisms through which an increase in circulating GCs mediate the downregulation of GRα mRNA and protein levels [81,82,83]. Several psychological and behavioural studies have linked an increase in GR gene methylation to a decrease in GRα mRNA and/or protein expression, in response to increases in circulating GCs [39,46,48,84,85]. Specifically, McCormick et al. [85] identified the promoter region of the exon variant 1_7_ as the region of the *Nr3c1* gene that undergoes the most significant methylation when rat pups experience ‘stressful’ conditions, with studies by Zhu et al. [46] and Niknazar et al. [39] demonstrating that this GC-mediated increase in the methylation status of exon 1_7_ promoter results in a decrease in GRα mRNA expression in adult Wistar rats. Supporting these findings, Mifsud et al. [84] demonstrated that an increase in circulating GCs, caused by acute stress, results in an increase in the methylation status of the exon 1_7_ promoter, which in turn, leads to a decrease in GRα mRNA expression of up to 75% in male Wistar rats. However, in contrast, Witzmann et al. [86] indicate that chronic, rather than acute stress, resulted in increased methylation of the promoter of exon 1_7_ in peripheral tissues. Moreover, exon 1_7_ methylation has also been shown to be important for regulation of the mouse *Nr3c1* gene, as an increase in the methylation status of this exon results in a reduction of GRα protein expression in mice [87]. 

Similarly, human studies demonstrate a negative correlation between DNA methylation of the *NR3C1* gene and GRα mRNA and protein expression [44,45,47,58,87]. For example, studies by McGowan et al. [45] and Wang et al. [47] correlate DNA methylation of exon 1_F_ of the human NR3C1 gene with a decrease in GRα mRNA expression in the brain hippocampus and PBMCs, respectively. Wang et al. [47] further relates this decrease in GRα mRNA expression to a decrease in GC sensitivity within PBMCs.

Although the studies discussed, focus on the methylation of the promoter of the GR exon variant, 1_7_, in rats, or its human homolog, exon 1_F_, it should be noted that the human GR exon 1_F_ (or 1_7_ in rats) was found to account for only 1–5% of total GR mRNA transcription within the brain [88] and peripheral tissues [89]. As such, the contribution of GR gene methylation at these sites to total GC-mediated downregulation of GRα may be negligible. Although other exon promoters, such as exon 1_B_, 1_C_ and 1_H_, have been shown to undergo GC-mediated methylation within the *NR3C1* gene, with an increase in the methylation status of these exon promoters relating to a reduction in GRα mRNA and/or protein expression [44,58,87], generally these other exons have been less investigated.

#### 3.1.2. Transcriptional Regulation

As previously described, the monomeric GC-GRα complex can transrepress genes through both an indirect tethering mechanism and by directly binding to a nGRE within the promoter of genes. Over 28 years ago, Burnstein et al. [90,91,92] postulated that the GR gene contains sequences which allow for the GC-mediated downregulation of GRα mRNA. In line with this postulation, Ramamoorthy et al. [68] identified the presence of an intragenic nGRE in exon 6 of the GR gene and subsequently demonstrated the GC-mediated regulation of GRα through a direct interaction of the receptor with this nGRE. Ramamoorthy et al. [68] went on to describe a GC-mediated autoregulatory loop that functions by inhibiting transcriptional initiation of the GR gene, consequently resulting in a decrease in nascent GRα mRNA expression of up to 90% in some cells such as A459 lung carcinoma cells. This transrepression of the GR gene is mediated via a long-range interaction between the GC-GRα complex at the nGRE and a NCOR1-deacetylase 3 (HDAC3)-containing repression complex, assembled at the transcriptional start site of the gene. The GC-dependent ability of GRα to regulate its own transcription was found to be consistent across human, mouse and rat cell lines, as well as in mouse tissues [68]. An increase in circulating GCs (whether endogenous or exogenous) is likely to augment this constitutive repression of the GR gene.

**Figure 3:**
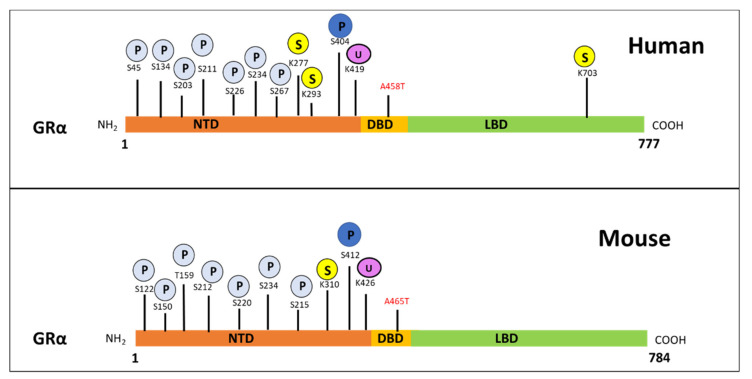
Post-translational modification sites for human (**top**) and mouse (**bottom**) GRα pertaining to phosphorylation, ubiquitination and sumoylation. The human GRα protein consists of 777 and the mouse GRα of 784 amino-acid residues, with both of these proteins undergoing PTMs at various sites. Phosphorylation (light blue) occurs primarily at serine residues while, sumoylation (yellow) and ubiquitination (purple) occur at lysine residues for both receptors. Moreover, phosphorylation at S404 in humans and S412 in mice (dark blue), as well as ubiquitination of K419 in humans and K426 in mice, is known to modulate receptor levels. The precise site of symolation which affect GRα stability, has not yet been determined. Indicated in red are the alanine to threonine point mutations which yield the dimerization deficient GR mutant, GRdim.

Interestingly, studies by Hua et al. [93,94] identify the sumoylation of GRα at lysine 293 (K293) in humans and K310 in mice (Figure 3), as a prerequisite for Dex-mediated GRα transrepression. The group illustrates the association of sumoylated and Dex-bound GRα with SMRT/NCOR1-HDAC3-containing repression complexes at nGRE sites within the promoters of genes, confirming that sumoylation of the GRα is indispensable in the above-described autoregulation of GRα transcription. Sumoylation is one of the many post-translational modifications (PTMs) which GRα undergoes and is described in more detail in an upcoming section.

#### 3.1.3. Post-Transcriptional Regulation

Post-transcriptional regulation results in the destabilization of the mature GRα mRNA transcript, rather than modulation of the levels of nascent GRα mRNA as is the case in transcriptional regulation of the GR gene. Destabilization of the mature GRα mRNA transcript is mediated by the incomplete base pairing of micro-RNAs (miRNAs) to adenylate uridylate (AU)-rich elements within the 3′-untranslated region (UTR) of the GRα mRNA transcript. The binding of these small (20–25 nucleotides), single-stranded non-coding RNAs, generally prevent the translation of the mRNA transcript and protein expression; however, similar to small interference RNAs (siRNAs), the binding of miRNAs could also result in the degradation of transcripts [95,96,97,98]. The downregulation of GRα mRNA mediated by miRNAs has been shown to occur across species [80,84,99,100,101] in response to an increase in circulating GCs, whether endogenous (disease-associated) or exogenous (treatment-associated), and is thus implicated in the development of acquired GC resistance.

As it pertains to rodent studies, four miRNAs (miR-96, miR-101a, miR-142-3p and miR-433p) have been shown to reduce GRα mRNA expression by up to 40% in mice exposed to adrenocorticotropic hormone (ACTH), which resulted in increased GC levels [99]. In addition, Mifsud et al. [84] demonstrated a stress related time-dependent increase in miR-124a that led to a reduction in GRα mRNA expression in male Wistar rats. Additionally, elevated levels of neuronally expressed miRNA-124-3p, was found to result in GRα downregulation, decreased GC sensitivity and an increase in depression-like symptoms in several rat and mouse studies [100,102].

Similarly, human studies negatively correlated miRNA-124-3p expression to GC sensitivity and GRα protein expression in ALL patients who display decreased sensitivity to GC therapy [101]. Additionally, the addition of miRNA-124-3p to a GC-sensitive cell line in vitro decreased the anti-proliferative and proapoptotic effect of GC therapy [101]. Similarly, elevated expression of miRNA-142-3p in ALL patients was also correlated with a decreased sensitivity to GC therapy [103].

Because miRNAs mainly function by blocking the translation of GRα mRNA transcripts, their effects are often only reflected at GRα protein level. This is demonstrated in studies by Vreugendenhil et al. [100] and Shimizu et al. [104] who investigated the effects of miR-18 and miR-124a on GRα mRNA expression in rats and mice, respectively. Both studies found that these miRNAs did not significantly affect GRα mRNA expression, but that GRα protein expression decreased.

### 3.2. GRα Protein Regulation

While GC-mediated regulation of GRα synthesis occurs, it is well established that GC-mediated regulation of the GRα pool occurs primarily via receptor protein degradation. This section will, thus, focus on GC-induced post-translational modifications of GRα, which induces receptor protein degradation, and the molecular mechanism through which degradation is mediated. While this section primarily reports on GRα protein degradation via the ubiquitin–proteasome system (UPS), it is important to note that GRα protein degradation also occurs via a lysosomal-dependent pathway. However, because there is no evidence to support a GC-mediated increase in the downregulation of GRα protein levels via lysosomal activity [105,106], this mode of receptor protein degradation has been omitted from the current review.

#### 3.2.1. Post-Translational Regulation

Once synthesized, GRα undergoes a number of PTMs which impact GC-responsiveness in selective tissues and may contribute to acquired GC resistance. The most important PTMs, which are directly involved in GRα protein turnover via the proteasome, are ubiquitination, phosphorylation and, less frequently, sumoylation (Figure 3) [16,107,108,109].

A number of amino acid sites are hyperphosphorylated in a GC-dependent manner during the PTM of the GRα [80]. Hyperphosphorylation of these sites is known to modulate GRα function as well as expression, however, for the purpose of this review only phosphorylation sites which affect GRα protein expression will be discussed. Specifically, the GC-mediated hyperphosphorylation of human GRα at Serine 404 mediates the receptor’s turnover, resulting in a decrease in GRα protein levels [110] and, therefore, plays an important role in GC sensitivity. Studies by Galliher-Beckley et al. [110] provides evidence that Dex-induced hyperphosphorylation, mediated by glycogen synthase kinase 3β (GSK3β), at Ser404 for the human GRα (and Ser412 for mouse GRα) (Figure 3), enhances the turnover of the receptor. Moreover, use of a mutant incapable of being phosphorylated at Ser404 or inhibition of phosphorylation at this site by a GSK3β inhibitor, 6-bromoindirubin-3′-oxime (BIO), confirmed that restricting Dex-induced Ser404 hyperphosphorylation of GRα resulted in an increase in receptor protein stability. Additionally, unliganded human GRα was found to undergo constant phosphorylation and dephosphorylation at Ser-203 and Ser-211, suggesting that, in the absence of ligand, different populations of phosphorylated GRα are present in vivo [111].

**Figure 4:**
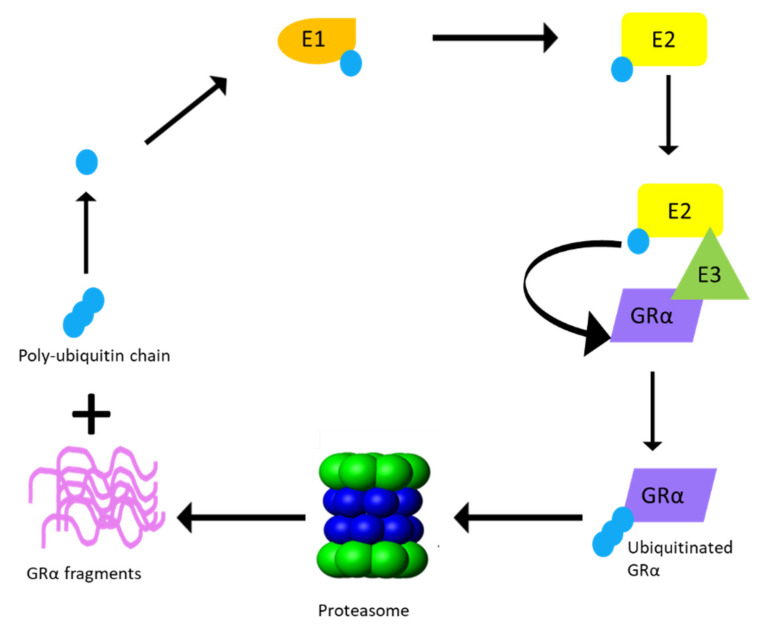
Proteasomal degradation of GRα via the ubiquitin–proteasomal system (UPS). Ubiquitination of the GRα requires multiple rounds of enzymatic processing before proteasomal degradation occurs. An activating enzyme, E1, activates ubiquitin and transfers it to a conjugating enzyme, E2. Hereafter, an E3 ligase from the RING finger family binds the GRα and recruits it to E2, which transfers the activated ubiquitin to K419 of the human GRα or K426 of the mouse GRα, until a poly-ubiquitin chain is formed. Once the poly-ubiquitin chain is complete, GRα is delivered to the proteasome and degraded, resulting in the release of GRα protein fragments and the poly-ubiquitin chain.

In order for the degradation of the receptor to take place following phosphorylation, GRα undergoes ubiquitination [61,66]. Previous studies have identified the single site for ubiquitination to be within the PEST (a peptide sequence that is rich in proline (P), glutamic acid (E), serine (S), and threonine (T) residues) degradation motif at lysine 426 (K426) in mice and lysine 419 (K419) in humans [61,66]. Furthermore, mutations at these specific residues restore GRα protein expression by restricting the downregulation of the receptor via the proteasome [61,66] (Figure 3). Once targeted for proteasomal degradation through phosphorylation, GRα is covalently linked at the specific lysine residue to a chain of ubiquitin molecules (polyubiquitin chain), which serves as a recognition motif for specific ubiquitin-binding subunits of the proteasome. The attachment of the 76-amino acid ubiquitin peptide to GRα requires the action of at least three sets of enzymes. Single ubiquitin peptides are first linked to E1 ubiquitin-activating enzymes before they are transferred to E2 ubiquitin-conjugating enzymes. The specificity of the final transfer of ubiquitin from the E2 enzyme is dependent on E3 ubiquitin-protein ligases, which provides specific substrate recognition. Two different classes of E3 ligases that use distinct mechanisms to bring about substrate-specific transfer of ubiquitin to the target proteins are involved. E3 ligases of the really interesting new gene (RING) finger family facilitate the transfer of ubiquitin from an E2 enzyme to the target protein, whereas homologous to the E6-AP C-terminus (HECT) domain, E3 ligases transfer a covalently linked ubiquitin that was acquired from an associating E2 enzyme [112]. Although the involvement of both RING finger and HECT domain E3 ligases have been described in the general proteasomal pathway, to date only RING finger E3 ligases have been shown to interact with the GRα (Figure 4).

In addition to phosphorylation and ubiquitination, GRα also undergoes sumoylation [93,107,109,113,114,115,116]. Sumoylation involves the covalent attachment of small ubiquitin-related modifiers (SUMO) to target proteins (i.e., GRα). While the sumoylation pathway shares many similarities with the UPS, there are distinct differences between the enzymes of these pathways. Specifically, the activation of SUMO by E1 activating enzymes is an ATP-dependent reaction, which results in the transference of SUMO to an E2 conjugative enzyme (in the case of GRα sumoylation the E2 enzyme is Ubc9), which subsequently attaches it to various lysine residues of the target protein.

While sumoylation is known to regulate the protein–protein and protein–DNA interactions of GRα, as well as receptor localization [93,107,109,113,114,115,116], one study specifically implicates the overexpression of the symoylation protein, SUMO-1, in the Dex-induced downregulation of GRα protein and provides evidence of proteasomal involvement in this downregulation [109]. In addition to being the only study to link sumoylation to GC-mediated GRα protein downregulation, the study also does not identify at which site (i.e., lysine residue) sumoylation occurs (Figure 3).

#### 3.2.2. UPS Enzymes That Modulate GRα Protein Levels

Various E2 conjugating enzymes and E3 ligases have been shown to interact with GRα to facilitate both ligand-dependent and ligand-independent regulation of receptor protein levels (Table 1). These UPS enzymes and their effect on GRα protein expression will be reviewed in the following section. Note that while the E3 ligase, E6-AP, has been shown to interact with GRα, its effect on GRα protein expression has not been determined; therefore, it will not be discussed [117,118].

The E2 conjugating enzyme, Ubiquitin-conjugating enzyme (UbcH7) has been shown to interact directly with the C-terminus of GRα in order to modulate the transactivation and protein expression levels of the receptor in a hormone-dependent manner [119]. Specifically, in the presence of Dex, an increase in the nuclear association of UbcH7 and GRα is observed, which results in a decrease in GRα protein levels due to GC-mediated downregulation of the receptor via the UPS [119]. Furthermore, Garside et al. [119] show that the overexpression of a dominant negative UbcH7 mutant, which is unable to transfer ubiquitin molecules, resulted in the increased stability of GRα and restricted the GC-mediated downregulation of the receptor via the UPS. The latter highlights the importance of UbcH7 in the ligand-induced downregulation of the GRα protein.

Tumor susceptibility gene 101 (TSG101) is an inactive E2 conjugating enzyme of the UPS that binds to unliganded, hypo-phosphorylated GRα. TSG101 resides in the cytoplasm and consists of a C-terminal coil domain, a central proline-rich segment and a N-terminal ubiquitin-conjugating (E2) variant domain [111,120]. The latter is, however, inactive due to the lack of a cysteine residue required to form a thioester bond with ubiquitin [111], while the coil domain interacts with the N-terminal AF-1 region of the GRα, which possess intrinsic transcriptional activation potential [111,120]. Experiments in which TSG101 was targeted for knockdown demonstrated a decrease in the stability of unliganded, hypophosphorylated GRα, thus revealing that TSG101 protects unliganded GRα from receptor downregulation [111]. Additionally, the study demonstrates the phosphorylation-dependent nature of the interaction between TSG101 and unliganded GRα, through the use of a human GRα phosphorylation mutant, GR(S203A/S211A). TSG101 was shown to have an increased association with GR(S203A/S211A), compared to its interaction with the unliganded wild type receptor. This was thought to be due to the lack of basal phosphorylation at S203/S211 of the mutant receptor. This is in stark contrast to the wild-type receptor, which constantly undergoes phosphorylation and de-phosphorylation cycles, even in its unliganded state. More recently, Wilkinson [121] reported on the importance of GRα phosphorylation status and its association with TSG101, by investigating the effect of the dimerization abrogating, non-steroidal GRα ligand, 2-(4-acetoxyphenyl)-2-chloro-N-methyl-ethylammonium chloride (CpdA), on the phosphorylation of human GRα at S404. As previously stated, the ligand-mediated hyperphosphorylation of S404 is associated with an increase in GRα protein downregulation. Wilkinson illustrates that CpdA treatment prevents the hyperphosphorylation of S404 which, in turn, resulted in the partial stabilization of the liganded receptor. Furthermore, considering the previously reported inhibition of S203 phosphorylation by CpdA [142] and the association and stabilization of hypophosphorylated GRα by TSG101, Wilkinson postulated that TSG101 association is important for the ability of CpdA to maintain GRα protein levels. Investigation into the possible link between CpdA-binding and TSG101 association in the stabilization of GRα revealed an increase in GRα protein expression following CpdA treatment in the presence of endogenous TSG101, but not following TSG101 knockdown experiments [121], suggesting that TSG101 is indeed required for the stabilization of GRα levels by CpdA.

F-box and WD repeat domain containing 7 alpha (FBXW7α) forms an SCF (Skp1/Cul1/F-box) type of E3 ubiquitin ligase complex that is known to target various proteins, including the GRα, for ubiquitination and proteasomal degradation [69,125,126]. The binding of this catalytically active E3 ligase requires the GC-mediated phosphorylation of the CDC4 phosphodegron motif of GRα, in order to mediate ubiquitination and proteasomal degradation [126,143]. Specifically, FBXW7α binding to GRα is dependent on the GSK3β-mediated phosphorylation of human GRα at Ser404, upon ligand binding [110]. Thus, ligand binding and the subsequent phosphorylation of Ser404, serves as a signal for ubiquitination and proteasomal degradation of GRα [125]. FBXW7α knockdown experiments, resulting in a significant increase in GRα protein levels in both HeLa and HEK293 cells, in a manner similar to the UPS inhibitor, MG132 [125], provides evidence for the role of FBXW7α in the GC-mediated downregulation of GRα. Furthermore, it was shown that a GRα phosphorylation mutant (Ser404A) was unable to undergo GC-mediated ubiquitination, which restricted the proteasomal degradation of the receptor [125]. Additionally, Wilkinson et al. [69] confirms, through the use of co-immunoprecipitation experiments, which were validated by proximity ligation assays (PLAs), that treatment with Dex and cortisol (F) resulted in an increase in the association of human GRα (hGRα) with FBXW7α. In contrast, unliganded or monomeric hGRα did not induce an interaction between hGRα and FBXW7α.

Another E3 ligase, the carboxy-terminus of Hsc70 interacting protein (CHIP), is a known modulator of basal nuclear receptor (NR) expression and availability, prior to ligand binding [144]. This E3 ligase binds to and decreases the ATPase activity of heat shock proteins (Hsps), the binding of which is known to stabilize and mediate the appropriate folding of NR proteins [145,146]. Thus, the CHIP-mediated disruption of the NR-Hsp interaction results in the misfolding and subsequent degradation of these NRs [127,131]. As it pertains to GRα, CHIP has been shown to ubiquitinate the receptor and directly target the GRα for degradation via the UPS [131]. Additionally, CHIP has been shown to bind to not only unliganded, but also liganded, GRα, thus acting independently of the phosphorylation status of the receptor [112,127]. Wang et al. [112] confirms a role for CHIP in mediating the GRα turnover of liganded GRα by showing enhanced GRα ubiquitination and subsequent degradation when CHIP is overexpressed. Moreover, Wilkinson [121] observed some GRα ubiquitination following treatment with dimerization abrogating ligand, CpdA, suggesting that monomeric GRα is capable of being ubiquitinated, however, it may not efficiently engage with the proteasome, thus evading degradation. Taking into account that monomeric GRα restricts the interaction of the receptor with the E3 ligase, FBXW7α, this demonstration of GRα ubiquitination following treatment with CpdA supports the notion of monomeric GRα, stabilizing an interaction with the other E3 ligases, such as CHIP. In support of this, Tateishi et al. [147] provides evidence that unliganded ER degradation is inhibited in cells where CHIP is absent, however, ligand-induced ER turnover in these cells still occurs. Therefore, Wilkinson [121] suggests that predominantly monomeric GRα associates with a complex involving CHIP and the catalytically inactive E2-conjugating enzyme, TSG101, whereas dimeric GRα, following treatment with dimerization promoting ligands such as Dex or F, preferentially associates with FBXW7α, rather than CHIP or TSG101.

In contrast to the other UPS enzymes, the E3 ligase, murine double minute 2 (Mdm2 or Hdm2, the human homologue) depends on the presence of p53 to form a trimeric complex with GRα, in order to mediate GRα protein turnover via the UPS. The GRα-p53-Mdm2/Hdm2 complex mediates GRα protein turnover independent of ligand binding [134]. More specifically, the interaction of GRα and p53 requires the ligase activity of Mdm2 [136]/Hdm2 [132] for the successful ubiquitination and proteasomal degradation of GRα. Sengupta et al. [132] demonstrated that Dex treatment enhances the association of GRα and p53, subsequently resulting in the increased downregulation of GRα. Additionally, the disruption of the interaction between p53 and Hdm2 inhibited the Dex-mediated ubiquitination of GRα and p53 within human umbilical endothelial cells, suggesting that the Dex-mediated downregulation of GRα requires the association of GRα with p53, the association of p53 with Hdm2 and the ligase activity of Hdm2. These results were confirmed by Kinyamu et al. [136], who found that both p53 and Mdm2 were required for estrogen-mediated GRα protein down-regulation via the proteasomal degradation pathway in MCF 7 M cells (MCF-7 cells which were stably transfected with mouse mammary tumor virus promoter-luciferase (MMTV-LUC) reporter and GRα expression constructs).

Pellino homolog-1 (Pellino-1) is a 47 kDa long protein which, due to its modulation of inflammatory signaling pathways, is considered a key component in inflammation, autoimmunity and tumorigenesis, with various studies indicating a positive correlation between its expression and grade of inflammation [148,149,150]. Due to the aforementioned, Petrillo et al. [137] investigated the role of Pellino-1 in the Dex-mediated reduction in GRα protein expression following β-arrestin-1 knockdown. The study noted that GRα associates with β-arrestin-1 in the cytoplasm in the absence of ligand and this complex formation persists after Dex treatment and translocation into the nucleus. Subsequent β-arrestin-1 knockdown experiments, however, revealed a time dependent reduction in GRα protein expression following Dex treatment in both MEF and human lung adenocarcinoma (A549) cells. The study then went on to confirm UPS involvement through PLA using antibodies directed at GRα and ubiquitin, respectively. PLA signals confirmed an increase in GRα ubiquitination in the absence of β-arrestin-1, following GRα activation by Dex [137]. An in vivo ubiquitination assay confirmed GRα ubiquitination to be poly-ubiquitination and not mono-ubiquitination with the former known to target proteins for proteasomal degradation, while the latter regulates protein trafficking [151]. Further qPCR results identified PELI1 as a novel GC-responsive gene, which is upregulated in the absence of β-arrestin-1. The PELI1 gene codes for the transcription of the Pellino-1 protein, which was found to exclusively interact with GRα in the absence of β-arrestin-1 [137]. The association of Pellino-1 with GRα significantly increased the Dex-mediated reduction in receptor levels through the addition of a K48-linked ubiquitin chain to GRα. The study concluded that Pellino-1 expression is upregulated in the absence of β-arrestin-1, allowing for the association of Pellino-1 to ligand-activated GRα, which subsequently enhanced receptor polyubiquitination and, thus, GRα proteasomal degradation [137].

Ubiquitin protein ligase E3 component N-recognin 1 (UBR1) belongs to a subset of E3 ligases, which specifically recognizes degradation signals within the N-terminal domain of proteins. These N-terminal signals are collectively referred to as N-degrons and include, but are not limited to, the polyubiquitylation of N-terminal lysine residues, which target the protein for proteasomal-dependent degradation [138,139,152]. Having previously demonstrated the role of UBR1 in the degradation of misfolded kinases upon Hsp90 inhibition [153], Sultana et al. [138] sought to determine the role of this E3 ligase in the degradation of the known Hsp90 client protein, GRα, upon Hsp90 inhibition. Specifically, the study illustrated the reduced rate of GRα protein degradation upon Hsp90 inhibition within UBR1 knockdown MEF cells, compared to wild type cells. Additionally, co-transfection experiments with rat UBR1 (rUBR1) and HA-tagged GRα (HA-GRα) indicated that rUBR1 overexpression resulted in a significant decrease in HA-GRα, even in the absence of Hsp90 inhibition [138], suggesting that GRα protein levels are sensitive to the expression of UBR1, independent of Hsp90 functionality. More recently, studies by Vu et al. [139] show an increase in both GRα mRNA and protein expression upon UBR1 knockdown, as well as the protein–protein association of UBR1 with a GRα isoform (GR-D), which primarily consists of the receptor LBD. However, following Hsp90 inhibition, GR-D underwent rapid degradation despite the total knockdown of UBR1 [139]. This suggests that UBR1 is not absolutely required for GRα downregulation upon loss of Hsp90-mediated protection, which is in agreement with previous studies that have demonstrated the modulation of NR expression by many proteolytical pathways [154]. Taken together, UBR1 facilitates the clearance of misfolded Hsp90 client proteins upon inhibition of the protein chaperone; however, cellular quality control mechanisms are able to function independently of its presence.

RING-type E3 ubiquitin transferase (RNF6) is known for its ubiquitination of various substrates, including the androgen receptor (AR) [155], transducin-like enhancer of split 3 (TLE3) [156] and the tyrosine phosphatase, SHP-1 [157], in order to mediate the proliferation of various types of cancer cells. The overexpression of RNF6 has also been linked to the development of doxorubicin resistance and, thus, leukemogenesis [158]. Since many multiple myeloma (MM) patients experience some degree of Dex resistance, a novel study by Ren et al. [140] sought to uncover a possible role for RNF6 in GC resistance in MM patients. The study found that RNF6 is overexpressed in various MM cell lines and that the presence of RNF6 increased the atypical K63-linked ubiquitination of GRα. RNF6 expression was found to stabilize unliganded GRα, even when receptor synthesis is inhibited by cycloheximide (CHX), increasing GRα half-life from 8 to 24 h, while RNF6 knockdown experiments resulted in a decrease in GRα protein levels [140]. Moreover, the study provides evidence that RNF6 binds to the LBD of the GRα and that its binding increases GRα transcriptional activity of prosurvival genes. MTT assays, furthermore, indicated that the ectopic expression of RNF6 significantly increased the viability of MM cells in the absence of Dex-treatment. Moreover, Dex treatment alone significantly decreased cell viability, an effect that was partially overturned by RNF6 expression. This suggests that the overexpression of RNF6 antagonizes the anti-proliferative action of Dex, with the latter describing a possible mechanism for GC resistance in MM [140].

Lastly, a recent study by Burke et al. [141] identified the E3 ligase, seven-in-absentia- mammalian homolog-2 (Siah2) as a modulator of GRα protein levels. Siah2 is a known regulator of fat mass and inflammation within adipose tissue [159,160] and has been shown to lead to the agonist-dependent activation of steroid receptors, such as GRα [156]. The study, which made use of either wild type mice (SIAH2+/+) or mice with a global deletion of SIAH2 (SIAH2-/-), found that corticosterone treatment led to an up to 42% decrease in GRα protein levels in SIAH2+/+ mice. In contrast, SIAH2-/- mice showed elevated levels of GRα protein, both in the absence and presence of corticosterone. Additionally, the overexpression of wild type Siah2 led to a significant reduction in GRα protein levels, an effect not evident in the presence of an enzymatically inactive mutant of the E3 ligase, suggesting that the enzymatic function of Siah2 is required for its modulation of GRα protein levels.

#### 3.2.3. Hsp90 as a Modulator of GRα Stability

**Figure 5:**
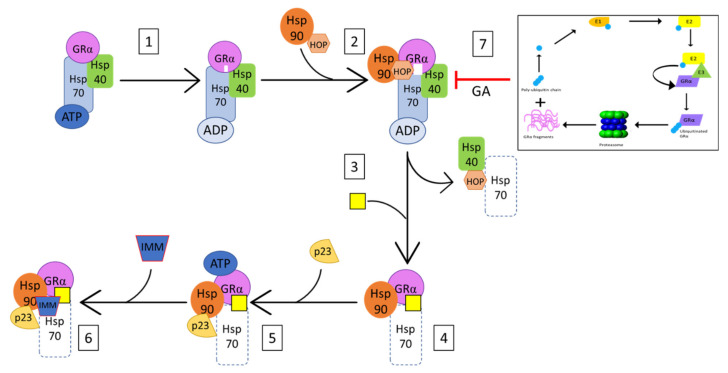
Simplified mechanism of GRα-Hsp90 association as adapted from Pratt et al. (145). (1) Hsp70 binds GRα in a Hsp40- and ATP-dependent manner, in order to facilitate the priming of the ligand-binding cleft (indicated in white) and the subsequent association of Hsp90 and Hop (2) Association of Hsp90 and Hop to the GRα-Hsp70 complex, results in the complete opening of the receptor’s ligand binding cleft (indicated in white), which allows for (3) the dissociation of Hsp40, Hop, as well as some of the Hsp70, and (4) the subsequent ligand-binding of the receptor (indicated by yellow rectangle). (5) Bound to Hsp90, GRα is now in its ATP-dependent conformation, allowing for the recruitment of p23, which stabilizes and prevents dissociation of the GRα-Hsp90 complex. (6) The dissociation of Hop at step 3 allows for the association of immunophilins (IMM) to the vacant tetratricopeptide repeat (TRP) acceptor site of bound Hsp90. Additionally, (7) Inhibition of GRα-Hsp90 heterocomplex formation, by GA results in the UPS-mediated degradation of GRα.

In addition to UPS enzymes, the protein chaperone, heat shock protein 90 (Hsp90), is known to play a significant role in the stabilization of GRα [145,161,162,163]. The interaction between unliganded-GRα and Hsp90 is highly ATP-dependent and initiated by Hsp70-Hsp90 organizing protein (HOP), a Hsp90 co-chaperone, and a GRα bound Hsp70 assembly complex (Figure 5). This heterocomplex facilitates the direct binding of Hsp90 to GRα, upon which the receptor becomes competent for hormone binding [162]. Following this transition, HOP, Hsp70 and several Hsp70 co-chaperones dissociate from the ligand-bound GRα-Hsp90 complex, allowing for the association of p23 and one of many tetratricopeptide repeat domain proteins (i.e., immunophilins (IMM)) (Figure 5). In the absence of Hsp90 machinery, the ligand binding clefts of GRα shifts between closed and open states, with the open state exposing hydrophobic residues of the receptor to the surrounding solvent. Prolonged exposure of these residues to the solvent may result in the unfolding and destabilization of the GRα, which in turn, results in the Hsp70-dependent ubiquitination of the receptor [145,146,164]. Additionally, studies by Fang et al. [162] demonstrate the interaction of ligand-bound GRα with Hsp90 within the nucleus, where Hsp90 binds GRα by imitating the interaction of the receptor with transcriptional coactivators. These studies establish Hsp90 as a stabilizer of both liganded and unliganded GRα. Furthermore, inhibition of the direct association of Hsp90 with GRα, though the use of geldanamycin (GA), has been shown to trigger the UPS-mediated degradation of GRα [138,165] (Figure 5).

## 4. GRα Downregulation and Receptor Conformation

While previous studies surrounding the impact of GRα dimerization on GRα signaling were mainly aimed at addressing the number of adverse side-effects which accompany GC therapy [166,167,168], rather than GC resistance, noteworthy reports have been made regarding the role of the GRα dimerization state on receptor turnover and, thus, in effect, the development of an acquired GC resistance.

Originally investigated for its role as a contraceptive [169,170], the SEGRAM CpdA has become a useful tool in the investigation of dimerization dependent GRα signaling. Not only was this GR modulator shown to bind and activate the GRα, but it was also shown to retain the anti-inflammatory activity of GRα signaling, while preventing many of the metabolic side effects which accompany GC therapy [25,26,27,171]. In vitro and in vivo studies into the molecular mechanism, through which CpdA mediates these effects, showed that, in contrast to the dimerization promoting, Dex, binding of the non-steroidal ligand inhibits dimerization of the GRα, effectively ensuring monomeric signaling of the receptor [25,172,173,174]. Additionally, unlike Dex, CpdA treatment was shown to maintain both GRα mRNA and protein levels [67,142,171,175], suggesting that the dimerization status of the receptor may influence the ligand-mediated regulation of the GRα. In agreement with the latter, Wilkinson et al. [69,121] showed that treatment with dimerization promoting GR agonists, Dex and F, resulted in an almost 50 h decrease in the half-life of GRα protein, compared to the unliganded receptor, which was shown to have a half-life of 70-h. In stark contrast, CpdA treatment did not affect the half-life of GRα protein. In fact, 72 h of CpdA treatment significantly increased GRα protein levels. Further investigation into the molecular mechanism of CpdA revealed that this GRα modulator prevents the GSK3β-mediated hyperphosphorylation of S404 of the human GRα. As previously stated, the hyperphosphorylation of this site by dimerization promoting ligands, such as Dex, has been shown to lead to GRα instability and subsequent degradation. Moreover, Wilkinson et al. [69,121] showed that the lack of S404 phosphorylation of the human GRα by CpdA restricts the interaction of the receptor with the E3 ligase enzyme, FBXW7α, inhibiting the subsequent degradation of the receptor via the proteasome [69,121].

As with CpdA, the restriction of GRα dimerization, through the use of the dimerization deficient GRα mutant, GRdim, has recently proven beneficial in the study of acquired GC-resistance [69,121,176]. Originally developed in 1994, this mutant receptor, which contains an Alanine to Threonine substitution in the dimerization loop of the DBD of the GR gene [177] (Figure 3), has been shown to have a restricted dimerization ability, compared to wild type GRα [178,179]. While the majority of GRdim-centered research focusses on improving the therapeutic index of GCs, with many illustrating this successfully [166,180,181], recent studies by Glantschnig et al. [176] and Wilkinson et al. [69,121] highlight the dimerization dependent downregulation of both GRα mRNA and protein. On a post-transcriptional level, Glantschnig et al. [176] found that Dex-activated GRα increased the expression of miR-29a which, in turn, downregulated GRα levels through the destabilization of GRα mRNA. Interestingly, the miR-29-mediated downregulation of GRα mRNA was abolished in the GRα dimerization deficient mouse embryonic fibroblasts (MEF-GRdim) cell line, suggesting, that the Dex-induced downregulation of GRα via miR-29a is dimerization dependent [176]. On a post-translational level, Wilkinson [69,121] shows that hGRwt protein undergoes significantly more Dex-induced downregulation, compared to hGRdim. Moreover, hGRdim protein was found to be unaffected by up to 72 h of Dex treatment, in stark contrast to hGRwt protein, which was downregulated in a time-dependent manner. Furthermore, Dex treatment was unable to induce the hyperphosphorylation of S404 in hGRdim; in fact, the receptor was completely void of even basal phosphorylation at this site. The lack of receptor phosphorylation restricted the association of GRdim with FBXW7α and inhibited the proteasomal degradation of the GRdim [69,121].

In addition to the abovementioned, restriction of GRα dimerization has also been shown to inhibit the repression of receptor gene transcription. More specifically, Ramamoorthy et al. [68], who reported on the Dex-mediated autoregulatory loop, through which the GRα negatively regulates its own transcription, notes that treatment with the GRα antagonist, mifepristone (RU486), does not induce the formation of the complex involved in the downregulation of GRα mRNA. Moreover, the study demonstrates no significant change in GRα mRNA levels following RU486 treatment, as well as restoration of GRα mRNA levels upon Dex and RU486 co-treatment, relative to Dex treatment alone. RU486, which has also been shown to be less effective in downregulating GRα protein levels [182], significantly restricts GRα dimerization, compared to Dex [183].

**Figure 6:**
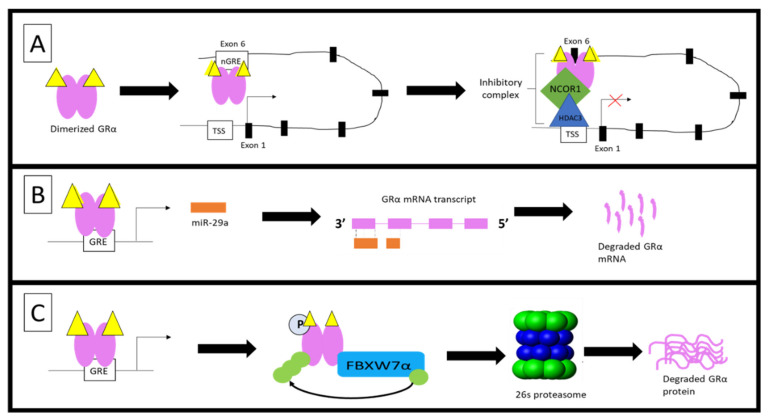
Dimerization-dependent regulation of GRα levels. Recent studies have revealed three mechanisms through which GRα dimerization increases receptor downregulation. (**A**) Ligand-induced dimerization enables the binding of GRα to a nGRE site located in exon 6 of the GR gene (NR3C1). Binding of GRα at this site, results in the recruitment of NCOR1 and HDAC3, which, due to chromatin looping, forms a repression complex at the transcription start site (TSS) of the gene, effectively, repressing GRα mRNA transcription [68]. (**B**) Dimeric GRα binds to a GRE site within the miR-29a promoter, resulting in the transcription of miR-29a. In turn, miR-29a associates with the 3′UTR region of the mature GRα mRNA transcript, resulting in the destabilization and degradation of GRα mRNA [176]. (**C**) Following ligand binding, hyperphosphorylated GRα interacts with the E3 ligase, FBXW7α, which tags the receptor for degradation by attaching ubiquitin molecules to specific lysine residues. GRα protein degradation occurs via the 26s proteasome [69,121].

Taken together, these studies identify three principal regulatory mechanisms through which dimerization promoting GRα agonists control receptor expression (Figure 6). A lack in our current understanding of the dimerization-dependent regulation of GRα levels, however, is the collective effect of these processes, as, to the best of our knowledge, no published study has investigated the combined transcriptional, translational and proteasomal involvement in the GC-mediated downregulation of GRα within an endogenous system. While the study by Wilkinson [69,121] accounts for translational and proteasomal regulation of GRα, it was unable to address transcriptional regulation due to the predominant use of transiently transfected receptors.

In a recent study, Parsonnete et al. [184] describes the physical association of both GRwt and GRdim protein with non-coding RNA hairpin structures via the DBD of the receptor. Additionally, Parsonnete et al. [184] illustrates that the GRwt binds to the RNA as a monomer and that the Gas5 hairpin RNA, an RNA repressor of the GR [185,186], competes for GRwt binding, with dsDNA containing a consensus GRE with a binding affinity that is in the same range. Furthermore, the study demonstrates that GRwt binds to DNA-GRE with a three-times higher affinity than GRdim, but with equal affinity for the non-coding RNA hairpin structure; thus, competition for receptor binding between RNA and DNA would favor the binding of GRdim to RNA rather than DNA.

Collectively, the findings by Parsonnete et al. [184] suggest a dimerization-independent association of GRα with non-coding RNA hairpin structures which may, in turn, suggest that a role for non-coding RNAs in the maintenance of GRdim protein stability, and effectively provide a mechanism of action to explain the significantly decreased ligand-mediated downregulation of GRdim protein, as compared to GRwt protein [69]. Tantalizingly, a recent unpublished study within our laboratory [187] demonstrated a significant increase in both the efficacy and potency of Dex to downregulate GRdim protein levels in the presence of the translational inhibitor, amanitin, compared to Dex treatment alone. Conversely, Dex and amanitin co-treatment resulted in an increase in GRwt protein levels, compared to Dex treatment alone. While these results may be attributed to the effects of miRNAs on the stability of GRα mRNA transcripts, miRNAs primarily mediate their effects by binding to the 3′UTR of mRNA transcripts [188], and as GRwt and GRdim only differ at a single amino-acid residue within the DBD [177], it is highly unlikely that miRNAs could differentiate between the mRNA transcripts of these receptors. A more likely occurrence is the stabilization of GRdim protein by non-coding RNAs.

### 4.1. Proposed Model for GC-Mediated GRα Regulation

**Figure 7:**
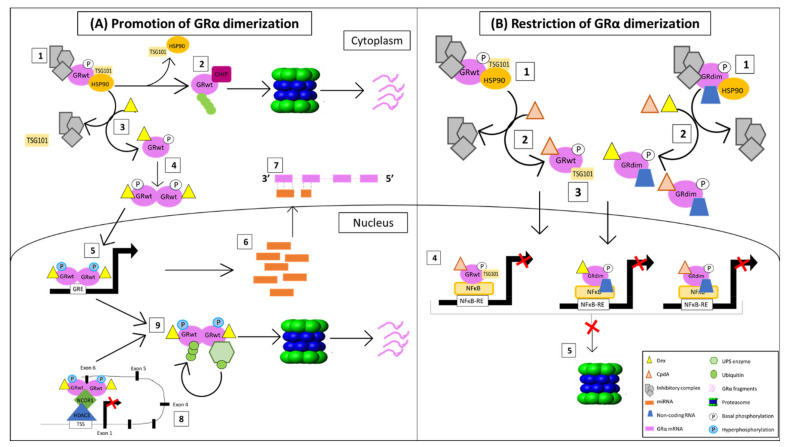
Current understanding of how GRα dimerization affects downregulation of the receptor. (**A**) Promotion of GRα dimerization and (**B**) Restriction of GRα dimerization. See text for detail.

Given the evidence provided by previous studies, as well as those highlighted within the study by Parsonnete et al. [184], we have constructed a proposed model, which briefly summarizes and addresses the most important findings regarding the ligand-mediated downregulation of GRα under conditions that promote GR dimerization (Figure 7A) and that restrict GRα dimerization (Figure 7B).

#### 4.1.1. Promotion of GRα Dimerization

The unliganded GRwt is primarily found within the cytoplasm, bound to an inhibitory complex [162,189], which contains, among other proteins, the inactive E2 conjugating enzyme TSG101 [111]. The latter is a UPS enzyme which specifically associates with the hypophosphorylated receptor, in order to inhibit the proteasomal degradation of the un-liganded receptor (Figure 7A point 1). However, upon Hsp90 dissociation, TSG101 is replaced by the E3 ligase CHIP, which targets the GRwt for proteasomal degradation through the addition of a poly-ubiquitin chain at a single lysine residue (K419 in humans and K426 in mice) [145,146] (Figure 7A point 2).

Dex binding to GRwt results in the dissociation of both the inhibitory complex [162] and TSG101 (Figure 7A point 3), after which the receptor dimerizes and translocates into the nucleus [174] (Figure 7A point 4). Once in the nucleus, the primarily nuclear GSK3β kinase [190,191] induces the hyperphosphorylation of dimeric GRwt at S404 in humans and S412 in mice [110], after which the homodimer binds and activates GRE-driven promoters in order to mediate the regulation of various GC sensitive genes [18,192] (Figure 7A point 5). This includes the transcription of miRNAs [80,84,99,100,101], which are then shuttled into the cytoplasm [193] (Figure 7A point 6). Once in the cytoplasm, these miRNAs bind to sequence-specific regions within the 3′UTR of the GRwt mRNA transcript, subsequently resulting in the destabilization and degradation of the receptor mRNA [95,96,97,98] (Figure 7A point 7). Additionally, in the nucleus, dimeric Dex-bound GRwt is able to bind to a nGRE site at exon 6 within the NR3C1 gene promoter in order to facilitate the autorepression of GR gene transcription via the formation of a NCOR1-HDAC3-repression complex at the transcription start site (TSS) of the gene [68] (Figure 7A point 8).

After mediating transcriptional regulation, the hyperphosphorylation at S404 in humans and S412 in mice allows for the association of GRwt with various UPS enzymes [69,119,121,125,126,132,133,134,135,136], which tags the receptor for proteasomal degradation via the association of a poly-ubiquitin chain at a single lysine residue (K419 in humans and K426 in mice) [66]. Classically, proteasomal degradation is thought to occur within the cyto-plasm [194], however, studies have provided evidence of the presence of the proteasome within the nucleus [195,196,197]. Thus, we believe that dimeric GRα undergoes proteasomal degradation within the nucleus following its transcriptional activity. The text continues here (Figure 7A point 9).

#### 4.1.2. Restriction of GRα Dimerization

Both unliganded GRwt and GRdim are primarily found within the cytoplasm, bound to an inhibitory complex [162]. However, while the complex of GRwt includes the E2 conjugating enzyme TSG101 [111], the current review postulates that the GRdim complex contains a non-coding RNA, which stabilizes the receptor protein [184] (Figure 7B point 1). Upon ligand binding, the receptors dissociate from the majority of their inhibitory complexes [162] (Figure 7B point 2). However, as they translocate into the nucleus, CpdA-bound GRwt remains associated with TSG101 [121], and GRdim remains associated with the stabilizing non-coding RNA. Moreover, CpdA binding does not induce dimerization of either GRwt nor GRdim [25,174]. Similarly, Dex-bound GRdim remains mostly monomeric [178] (Figure 7B point 4). Once in the nucleus, the monomeric receptors primarily mediate the transrepression of various genes (i.e., IL-6) by disrupting DNA-bound TFs (i.e., NFκB) [20] (Figure 7B point 4). Following gene regulation, neither CpdA-bound GRwt nor Dex/CpdA-bound GRdim undergo UPS-targeted degradation due to lack of receptor hyperphosphorylation at S404 in humans and S412 in mice, which is required for the association of the receptor with UPS enzymes [69,121] (Figure 7B point 5).

## 5. Conclusions

Acquired GC resistance as a consequence of GC-mediated downregulation of the GRα, results in various degrees of tissue specific GC insensitivity and presents an ever-increasing therapeutic challenge for the chronic use of GCs. While it has long been known that GCs regulate the GRα pool at several levels and through various molecular mechanisms, recent studies have elucidated the dimerization-dependent nature of some of these regulatory processes [68,69,121,176,187]. In doing so, the importance of GRα conformation in addressing the two major pitfalls of chronic GC-therapy, namely, the generation of side-effects and the development of acquired GC resistance [23,68,69,174,175,176], has been highlighted. Specifically, in the treatment of chronic inflammatory conditions, these studies provide a wealth of evidence in support of the usage of conformationally biased ligands, which preferentially induce the GRα monomeric state over receptor dimerization [15,21]. While this strategy may not be completely void of adverse side-effects, the risk of chronic hyperglycemia [25,26,27,171], and the accompanying risk of cardiovascular disease and metabolic syndrome, could be avoided. Moreover, as illustrated by recent studies, the induction of GRα monomeric signaling may preserve GC-sensitivity, which is especially at risk during chronic GC treatment [68,121,176]. However, while these ligands may be a viable avenue to explore in the development of more tailored GC-treatment regimes, the impact of GR dimerization on receptor signaling, by and large, remains a mystery. Louw [21] suggests a quantitative analysis of GRα dimerization bias, in order to accurately measure and quantify the impact of receptor dimerization. This is especially important considering the fact that not all GC-mediated therapeutic effects are mediated via monomeric GRα-signaling, just as, not all GC-mediated side-effects are mediated via dimeric GRα-signaling [24,198,199]. Furthermore, considering the multitude of cellular processes that are directly or indirectly affected by GRα signaling, investigations into the impact of GRα dimerization on receptor signaling should include a systems biology approach, such as that followed by Somvanshi et al. [200] where investigators developed a mathematical model of the HPA-axis and immune system in order to explore the link between inflammation and a hyperactive HPA-axis in post-traumatic stress disorder (PTSD). The use of a mathematical model allowed researchers to account for the multiple feedback mechanisms between the HPA-axis and the immune system and to come to the counterintuitive conclusion that increased GR sensitivity may augment, rather than diminish, inflammation in PTSD.

## Figures and Tables

**Table 1:** UPS enzymes known to interact with the GRα.

Name	Type of UPS Enzyme	GRα Ligand-Binding Status	GRα Phosphorylation Status	Effect on GRα Protein Level	References
UbcH7	E2 conjugating enzyme	Liganded	Hyperphosphorylated	Reduce	[119]
TSG101	Inactive E2 conjugating enzyme	Unliganded	Hypophosphorylated	Stabilize	[111,120,121]
FBXW7α	E3 ligase (RING finger family) [122,123,124]	Liganded	Hyperphosphorylated	Reduce	[69,121,124,125,126]
CHIP		Ligand-independent	Phosphorylation-independent	Reduce	[112,124,127,128,129,130,131]
Mdm2/Hdm2		Ligand-independent but requires p53	Phosphorylation-independent	Reduce	[124,132,133,134,135,136]
Pellino-1		Liganded	Hyperphosphorylated	Reduce	[137]
UBR1		Unliganded	Hypophosphorylated	Reduce	[124,138,139]
RNF6		Ligand-independent	Phosphorylation-independent	Stabilize	[140]
Siah2		Ligand-independent	Hyperphosphorylated	Reduce	[141]
